# Long‐term cardiovascular risk in women with hypertensive disorders of pregnancy: Insights from polygenic risk scores

**DOI:** 10.1111/aogs.70021

**Published:** 2025-07-31

**Authors:** Anna Kivioja, Jaakko Tyrmi, Elli Toivonen, Heini Huhtala, FinnGen, Tiina Jääskeläinen, Johannes Kettunen, Tanja Saarela, Hannele Laivuori

**Affiliations:** ^1^ Faculty of Medicine and Health Technology, Center for Child, Adolescent, and Maternal Health Research Tampere University Tampere Finland; ^2^ Department of Obstetrics and Gynecology Kanta‐Häme Central Hospital, the Wellbeing Services County of Kanta‐Häme Finland; ^3^ Systems Epidemiology, Research Unit of Population Health, Faculty of Medicine University of Oulu and Biocenter Oulu Oulu Finland; ^4^ Department of Obstetrics and Gynecology Tampere University Hospital, the Wellbeing Services County of Pirkanmaa Finland; ^5^ Faculty of Social Sciences Tampere University Tampere Finland; ^6^ Department of Food and Nutrition University of Helsinki Helsinki Finland; ^7^ Medical and Clinical Genetics University of Helsinki and Helsinki University Hospital Helsinki Finland; ^8^ Department of Clinical Genetics Kuopio University Hospital, Wellbeing Services County of North Savo Finland; ^9^ Institute for Molecular Medicine Finland University of Helsinki Helsinki Finland

**Keywords:** cardiovascular disease, hypertensive disorders of pregnancy, polygenic risk score, preeclampsia

## Abstract

**Introduction:**

The association between preeclampsia (PE) and an elevated risk of cardiovascular disease (CVD) is well documented. Recent genome‐wide association studies of PE have further highlighted their possible common genetic background. We investigated how the history of hypertensive disorders of pregnancy (HDP), including the PE phenotype, and normotensive pregnancy, combined with polygenic risk scores (PRSs) for PE (PE‐PRS), high systolic blood pressure (SBP‐PRS), coronary artery disease (CAD‐PRS) or stroke (stroke‐PRS), affects the risk for CVD.

**Material and Methods:**

The study was conducted in the FinnGen cohort of 213 942 Finnish women, including 8858 women with PE, 17916 women with any HDP, and 196 026 parous controls. PE women were included in the HDP phenotype. Participants were classified based on their PRSs into three groups: low (<20%), moderate (20–80%) and high (>80%) genetic risk. Women with normotensive pregnancies and moderate PRSs served as controls.

**Results:**

Women with PE and a high genetic risk for PE, high SBP, CAD, and stroke had a significantly increased risk of CVD compared to women with normotensive pregnancies and a moderate genetic risk. The hazard ratios (HRs) for CVD were 1.87 for PE‐PRS, 2.31 for SBP‐PRS, 1.94 for CAD‐PRS, and 2.07 for stroke‐PRS, all *p*‐values 2 × 10^−16^. A similar pattern was observed in women with any HDP. Among women with normotensive pregnancies, a high genetic risk led to only a modest increase in CVD risk. The corresponding HRs were 1.07 for PE‐PRS (*p* = 5 × 10^−5^), 1.32 for SBP‐PRS, 1.19 for CAD‐PRS, and 1.16 for stroke‐PRS (all *p* values 2 × 10^−16^). Across all four PRSs, the impact of PE and any HDP on CVD risk was greater than that of genetic risk alone. The elevated CVD risk persisted up to the age of 80.

**Conclusions:**

In women with PE or any HDP, a high genetic risk for PE, high SBP, CAD, and stroke further increases the overall risk of CVD. Among women with normotensive pregnancies, a high genetic risk confers only a modest increase in CVD risk. In evaluating long‐term CVD risk, clinical risk assessment, including obstetric history, appears to outperform genetic risk evaluation using PRSs.

## Graphical abstract

FinnGen data of 213 942 women was utilized in order to investigate the risk of cardiovascular disease in women with and without hypertensive disorders of pregnancy and with diverse genetic risks for cardiovascular disease and preeclampsia measured with polygenic risk scores.

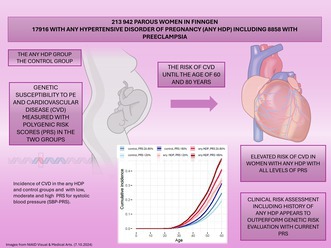

**Abbreviations**
PE: preeclampsia
HDP: hypertensive disorder of pregnancy
CVD: cardiovascular disease
GWAS: genome‐wide association study
BMI: body mass index
PRS: polygenic risk score
SBP: systolic blood pressure
CAD: coronary artery disease
CI: confidence interval

**Key message**

Hypertensive disorders of pregnancy increase a woman's risk for cardiovascular disease until elderly. A high genetic risk for preeclampsia and cardiovascular diseases further elevates the risk; however, the role of hypertensive disorders of pregnancy appears to be more relevant.

## 1 INTRODUCTION

Preeclampsia (PE) is a hypertensive disorder of pregnancy (HDP) affecting 3–5% of pregnancies in developed countries.^1^ PE is known to be associated with an elevated risk of cardiovascular disease (CVD) in later life,^2^, ^3^, ^4^ and further, CVD remains one of the leading causes of death in women.^5^, ^6^ Increased risk of chronic hypertension,^7^, ^8^, ^9^ ischemic heart disease^9^ and cerebrovascular disease^7^, ^9^ has been documented in previous studies in women with a history of PE. In recent genome‐wide association studies (GWAS) studies certain risk alleles for high blood pressure, kidney function and body mass index, have been associated with PE.^10^, ^11^ However, the evidence on common genetic factors affecting the onset of CVD in concert with clinical risk factors, is scarce.

In the past decade, GWASs have generated a large number of genetic associations for various diseases and traits.^12^ This advancement has enabled utilization of polygenic risk scores (PRSs) in medical research.^13^ PRS is formed as a weighted sum of risk alleles found in GWAS studies to be associated with the disease.^14^ It describes an individual's overall genetic risk to a trait or disease. So far, PRSs have mostly been utilized in research, but the possible role of PRS in clinical practice has been investigated.^13^, ^15^ In the future, they might help in identifying individuals with a high genetic risk in need of a more intensive follow‐up.^15^, ^16^

We hypothesized that women with a history of HDP (including also women with PE and PE with severe symptoms) and a high PRS for PE (PE‐PRS), high systolic blood pressure (SBP‐PRS), coronary artery disease (CAD‐PRS) or stroke (stroke‐PRS) have an independent association with an increased risk for CVD compared to women with a history of normotensive pregnancies and similar genetic risk. Therefore, we aimed to determine the risk of CVD in the groups of women with PE, any HDP, PE with severe symptoms, and normotensive pregnancies in three PRS categories of low (PRS <20th percentile), moderate (PRS 20th–80th percentile) and high risk (PRS >80th percentile) until 60 and 80 years of age.

## 2 MATERIAL AND METHODS

### 2.1 Cohort description

**FIGURE 1:**
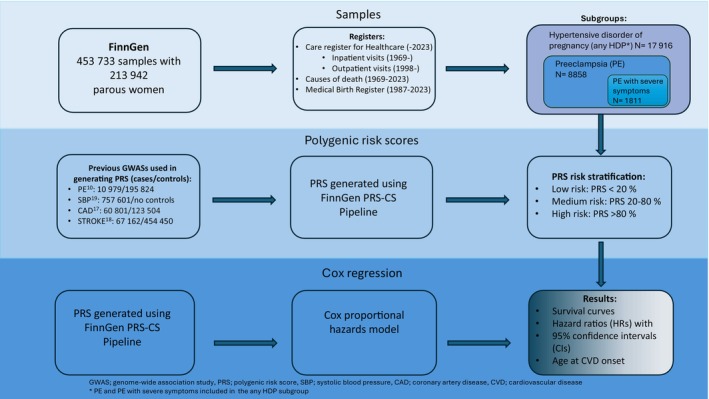
Workflow chart demonstrating the materials and methods of study. Expanded chart detailing case/control International Classification of Diseases (ICD)‐codes available in Figure S1.

The study phenotypes in FinnGen were based on codes of the 10th, 9th, and 8th revisions of the International Classification of Diseases (ICD) (Figure S1). PE women were also included in any HDP phenotype, as illustrated in Figure 1 and more detailed in Figure S1. The FinnGen participants are of Finnish ancestry. The genotype data were obtained from Finnish nationwide biobanks that are then linked with digital health records from the Care Register for Healthcare (inpatient register from 1969 onwards, outpatient register from 1998‐2023), Causes of Death (1969‐2023) and Medical Birth register (1987‐2023). The data were collected until April 2023. The Data Freeze 11 used in this study contains the genomic and health record data for 213 942 women. A total of 8858 PE and 17 916 any HDP cases were included (PE women were included in the HDP phenotype) with 196 026 controls. Out of 8858 PE women, 1811 women had PE with severe symptoms (Figure 1). In order to identify HDP subtype‐related factors associated with the CVD risk, we chose to examine those with PE with severe symptoms separately.

Patients and control subjects in the FinnGen study provided informed consent for biobank research, based on the Finnish Biobank Act. Alternatively, older research cohorts, collected prior to the start of FinnGen (in August 2017), were collected based on study‐specific consents and later transferred to the Finnish biobanks after approval by Fimea, the National Supervisory Authority for Welfare and Health. Recruitment protocols followed the biobank protocols approved by Fimea. The Coordinating Ethics Committee of the Hospital District of Helsinki and Uusimaa (HUS) approved the FinnGen study protocol Nr HUS/990/2017.

### 2.2 Genotyping

DNA isolation was carried out separately in each of the nine Finnish biobanks that provided samples for the FinnGen project. The following DNA extraction kits were used according to the manufacturers' instructions: Chemagic DNA Blood 250 and 400 (PerkinElmer), NucleoSpin 96 Tissue Core Kit (Macherey‐Nagel), QIAsymphony DSP DNA Midi Kit (QIAGEN), and sbeadex Blood DNA Purification Kit (LGC, Biosearch Technologies). Sample genotyping in FinnGen was performed using Illumina and Affymetrix arrays (Illumina Inc., San Diego, and Thermo Fisher Scientific, Santa Clara, CA, USA) at the Institute for Molecular Medicine Finland (FIMM) Technology Centre, University of Helsinki. Genotype calls were made using GenCall or zCall for Illumina and the AxiomGT1 algorithm for Affymetrix data. Genotypes with a Hardy–Weinberg equilibrium (HWE) *p*‐value of *p* < 1 × 10^−6^, a minor allele count of <3, and a genotyping success rate of <98% were removed. Samples with ambiguous sex, high genotype missingness (>5%), and those that were outliers in population structure (>4 SD from the mean on the first two dimensions) were omitted. Samples were pre‐phased with Eagle 2.3.5 using 20,000 conditioning haplotypes, and genotypes were imputed with Beagle 4.1 using the SiSu v4 imputation reference panel.

### 2.3 Polygenic Risk Scores

PRSs were calculated based on earlier large GWAS studies^17^, ^18^, ^19^ obtained from the GWAS catalogue.^20^ FinnGen PRS pipeline (detailed in https://github.com/FINNGEN/CS‐PRS‐pipeline) was then used to create the allele weights for polygenic risk scores. In brief, the pipeline first obtains a set of 1.1 million genetic variants from the European linkage disequilibrium reference panel with 1 derived from samples from the 1000 Genomes Project. PRS‐CS software^21^ is then used to recalculate single‐nucleotide polymorphism weights from GWAS summary statistics and a linkage disequilibrium reference panel by utilizing a Bayesian regression framework and placing continuous shrinkage priors on single‐nucleotide polymorphism effect sizes. PRS for each trait is then calculated by applying the weights for FinnGen samples using Plink software version 1.9.^22^

### 2.4 Study groups and settings

Groups of women with PE, any HDP, and PE with severe symptoms were divided into three groups based on their PRSs: low risk (<20th percentile), moderate risk (20th–80th percentiles) and high risk (>80th percentile). For each study group (PE, any HDP, PE with severe symptoms) we formed a control group as illustrated in Figure S1. Control women with moderate PRS served as a reference group for each of the study groups.

Women with pre‐existing CVD diagnosis before hypertensive pregnancy were excluded, as well as women with obstetric diagnoses indicating a prior CVD as presented in Figure S1. In the first section, we performed the analyses including chronic hypertension as an outcome. However, as chronic hypertension may be widely underdiagnosed before and after pregnancy, we performed these analyses also excluding chronic hypertension as an outcome. We observed the incidence of CVD until the age of 60 and 80 years.

### 2.5 Statistical analyses

A Cox proportional hazards model was employed to estimate survival curves, hazard ratios (HRs), and 95% confidence intervals (CIs). The proportional hazards assumption in our models was confirmed using Schoenfeld residuals and log–log inspection. The models were adjusted for birth year, genotyping array, cohort, body mass index, and the first 10 genetic principal components of ancestry.

The mean age at CVD onset was estimated among individuals with and without PE and any HDP, stratified by PRS categories. Pairwise comparisons were performed against the control group with moderate PRS (20–80%) using two‐sided *z*‐tests. Groupwise differences and their statistical significance were visualized using forest plots.

Statistical analyses were performed using R v. 4.4.0.

## 3 RESULTS

**FIGURE 2:**
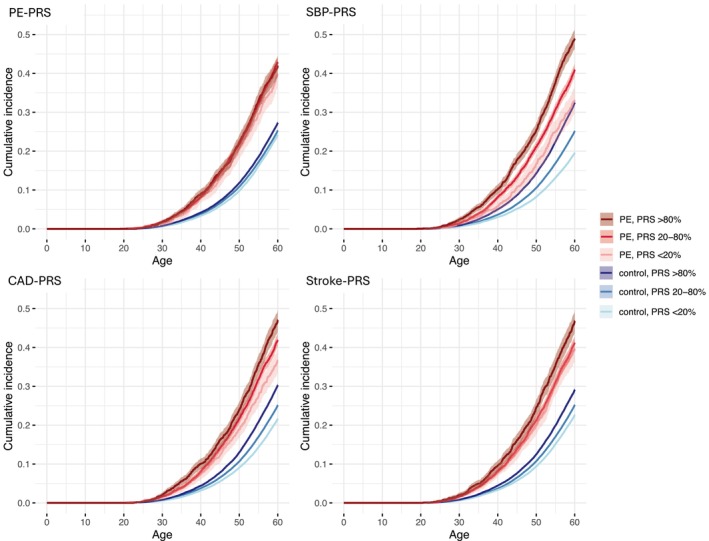
Cumulative events of cardiovascular disease (CVD) until 60 years of age presented in women with preeclampsia (PE) and control women stratified by polygenic risk scores (PRSs) for PE, systolic blood pressure (SBP), coronary artery disease (CAD) and stroke. PE and control women are divided into three groups according to their PRSs: Low (<20th percentile), moderate (20th–80th percentiles), high (>80th percentile).

**FIGURE 3:**
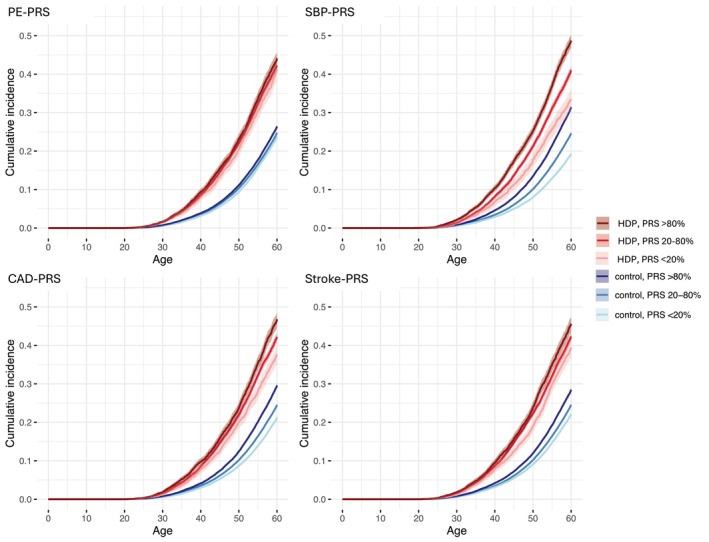
Cumulative events of cardiovascular disease (CVD) until 60 years of age presented in women with hypertensive disorders of pregnancy (any HDP) and control women stratified by polygenic risk scores (PRSs) for preeclampsia (PE), systolic blood pressure (SBP), coronary artery disease (CAD) and stroke. Any HDP and control women are divided into three groups according to their PRSs: Low (<20th percentile), moderate (20th–80th percentiles), high (>80th percentile). Women with PE were included in the any HDP group.

Until the age of 60, women with PE and a high PRS showed an increased risk of CVD compared to women with normotensive pregnancies and a moderate PRS: PE‐PRS, HR 1.87 (*p*‐value 2 × 10^−16^), SBP‐PRS 2.31 (2 × 10^−16^), CAD‐PRS 1.94 (2 × 10^−16^), stroke‐PRS 2.07 (2 × 10^−16^). See Tables 1 and 2, Figures 2 and 3. The risk of CVD was similarly elevated in women with any HDP and with high, respective PRSs: PE‐PRS, HR 2.06 (*p*‐value 2 × 10^−16^), SBP‐PRS 2.39 (2 × 10^−16^), CAD‐PRS 2.12 (2 × 10^−16^), stroke‐PRS 2.09 (2 × 10^−16^).

**TABLE 1:** The risk of cardiovascular disease (CVD) in the study groups (hypertensive disorder of pregnancy (any HDP), preeclampsia (PE) and PE with severe symptoms) reported as hazard ratios (HR) with 95% confidence intervals (CIs).

Study phenotype	Any hypertensive disorder of pregnancy, *n* = 17 916	*p*‐value	Preeclampsia, *n* = 8858	*p*‐value	Preeclampsia with severe symptoms, *n* = 1811	*p*‐value
PRS % group	HR (95% CI)		HR (95% CI)		HR (95% CI)	
Preeclampsia						
<20	1.77 (1.62–1.93)	2 × 10^−16^	1.63 (1.44–1.84)	3 × 10^−7^	2.24 (1.77–2.84)	3 × 10^−11^
20–80	1.90 (1.82–1.99)	2 × 10^−16^	1.82 (1.72–1.94)	2 × 10^−16^	2.13 (1.88–2.41)	2 × 10^−16^
>80	2.06 (1.93–2.20)	2 × 10^−16^	1.87 (1.71–2.04)	2 × 10^−16^	2.06 (1.70–2.50)	9 × 10^−14^
Systolic blood pressure						
<20	1.48 (1.33–1.64)	5 × 10^−13^	1.37 (1.19–1.57)	9 × 10^−6^	1.76 (1.33–2.33)	8 × 10^−5^
20–80	1.81 (1.73–1.90)	2 × 10^−16^	1.72 (1.62–1.83)	2 × 10^−16^	2.18 (1.93–2.47)	2 × 10^−16^
<80	2.39 (2.26–2.53)	2 × 10^−16^	2.31 (2.13–2.50)	2 × 10^−16^	2.33 (1.95–2.77)	2 × 10^−16^
Coronary artery disease						
<20	1.71 (1.57–1.87)	2 × 10^−16^	1.63 (1.45–1.84)	5 × 10^−16^	1.80 (1.36–2.37)	3 × 10^−5^
20–80	1.93 (1.84–2.02)	2 × 10^−16^	1.81 (1.71–1.93)	2 × 10^−16^	2.25 (2.00–2.54)	2 × 10^−16^
>80	2.12 (1.99–2.27)	2 × 10^−16^	1.94 (1.78–2.13)	2 × 10^−16^	2.08 (1.71–2.52)	1 × 10^−13^
Stroke						
<20	1.78 (1.64–1.93)	2 × 10^−16^	1.68 (1.50–1.88)	2 × 10^−16^	2.01 (1.60–2.52)	2 × 10^−9^
20–80	1.92 (1.83–2.01)	2 × 10^−16^	1.75 (1.64–1.86)	2 × 10^−16^	2.04 (1.80–2.32)	2 × 10^−16^
>80	2.09 (1.96–2.24)	2 × 10^−16^	2.07 (1.89–2.27)	2 × 10^−16^	2.52 (2.08–3.06)	2 × 10^−16^

*Note*: PE women were included in the any HDP group. Groups were stratified by polygenic risk score (PRS) for PE, systolic blood pressure, coronary artery disease, and stroke, to percentiles (low <20th percentile, moderate 20th–80th percentile, high >80th percentile). Control women with moderate PRS served as a reference group. The models were adjusted for birth year, genotyping array, cohort, body mass index and the first 10 genetic principal components of ancestry.

Abbreviations: CI; confidence interval, HR; hazard ratio, PRS; polygenic risk score.

**TABLE 2:** Number of women in the study—and control groups and in the groups of low (<20th percentile), moderate (20th–80th percentile) and high (>80th percentile) polygenic risk score (PRS) for preeclampsia, high systolic blood pressure, coronary artery disease, and stroke.

Study phenotype PRS % group	Any hypertensive disorder of pregnancy, *n* = 17 916/196026		Preeclampsia, *n* = 8858/205084		Preeclampsia with severe symptoms, *n* = 1811/212131	
	Cases	Controls	Cases	Controls	Cases	Controls
Preeclampsia						
<20	2812	39 977	1336	41 453	256	42 533
20–80	10 522	117 842	5231	123 133	1074	127 290
>80	4582	38 207	2291	40 498	481	42 308
Systolic blood pressure						
<20	2135	40 654	1174	41 615	232	42 557
20–80	10 275	118 089	5205	123 159	1046	127 318
>80	5506	37 283	2479	40 310	533	42 256
Coronary artery disease						
<20	2905	39 884	1519	41 270	280	42 509
20–80	10 793	117 571	5289	123 075	1092	127 272
>80	4218	38 571	2050	40 730	439	42 350
Stroke						
<20	3135	39 654	1581	41 208	322	42 467
20–80	10 687	117 677	5258	123 106	1070	127 294
>80	4094	38 695	2019	40 770	419	42 370

*Note*: The models were adjusted for birth year, genotyping array, cohort, body mass index and the first ten genetic principal components of ancestry.

Abbreviation: PRS, polygenic risk score.

Until 80 years of age, women with a history of PE or any HDP represented a higher risk of CVD in all levels of PRSs for PE, SBP, CAD, and stroke compared to women with a history of normotensive pregnancies and moderate respective PRSs (Tables S1 and S2). The risk seemed mildly evened from 60 to 80 years of age.

When we excluded chronic hypertension from the CVD outcomes, the similar trend remained; those women with a history of PE and HDP showed an elevated risk for CVD in all three PRS categories and with all tested PRSs. However, the association was attenuated (Table S1).

In the subgroup of women with a history of PE with severe symptoms, the risk for CVD seemed further elevated (Table 1). Due to a small number of women in this subgroup, the comparisons between the three PRS groups could not be performed.

In normotensive women, the risk for CVD seemed slightly elevated in those with high PRS with all four tested PRSs (Table 3). Normotensive women reached the same level of CVD risk as those with a history of PE only when we categorized SBP‐PRS groups into the top 1%, middle 98%, and bottom 1%. In this revised grouping, normotensive women in the top 1% presented an HR of 1.77 (CI 1.61–1.94), similar to those in the middle 98% of the PE group, with an HR of 1.77 (CI 1.69–1.86). With a high genetic risk for CVD (PRS >80th percentile) and normotensive pregnancies, the risk of CVD does not appear to increase substantially compared to those women with a moderate genetic risk until 80 years of age (HR 1.04–1.17) (Table S2).

**TABLE 3:** The risk of cardiovascular disease (CVD) in women with normotensive pregnancies reported as hazard ratios (HR) with 95% confidence intervals (CIs).

Normotensive *n* = 196 026			
PRS % group	*N*	HR (95% CI)	*p*‐value
Preeclampsia			
<20	39 977	0.97 (0.94–1.00)	0.08
20–80	117 842	1	
>80	38 207	1.07 (1.03–1.10)	5 × 10^−5^
Systolic blood pressure			
<20	40 654	0.79 (0.76–0.82)	2 × 10^−16^
20–80	118 089	1	
>80	37 283	1.32 (1.29–1.36)	2 × 10^−16^
Coronary artery disease			
<20	39 884	0.89 (0.86–0.92)	1 × 10^−12^
20–80	117 571	1	
>80	38 571	1.19 (1.15–1.22)	2 × 10^−16^
Stroke			
<20	39 654	0.91 (0.88–0.94)	3 × 10^−8^
20–80	117 677	1	
>80	38 695	1.16 (1.13–1.20)	2 × 10^−16^

*Note*: Women were stratified by polygenic risk scores (PRSs) for preeclampsia, systolic blood pressure, coronary artery disease, and stroke, to percentiles (low <20th percentile, moderate 20th–80th percentile, high >80th percentile). Women with moderate PRS (20–80th percentile) served as a reference group. The models were adjusted for birth year, genotyping array, cohort, body mass index and the first ten genetic principal components of ancestry.

Abbreviations: CI, confidence interval; HR, hazard ratio; PRS, polygenic risk score.

**FIGURE 4:**
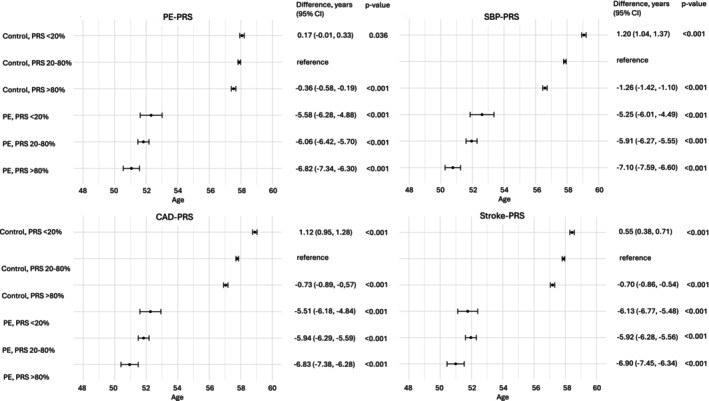
Mean age at cardiovascular disease (CVD) onset across preeclampsia (PE) and control groups stratified by polygenic risk score (PRS) percentiles. Error bars indicate 95% confidence intervals. *p*‐values reflect two‐sided comparisons against the control group with mid‐range PRS (20–80%). PE and control women were divided according to PRSs for PE (PE‐PRS), systolic blood pressure (SBP‐PRS), coronary artery disease (CAD‐PRS) and stroke (stroke‐PRS) in the groups of low (<20th percentile), moderate (20th–80th percentile) and high risk (>80th percentile).

**FIGURE 5:**
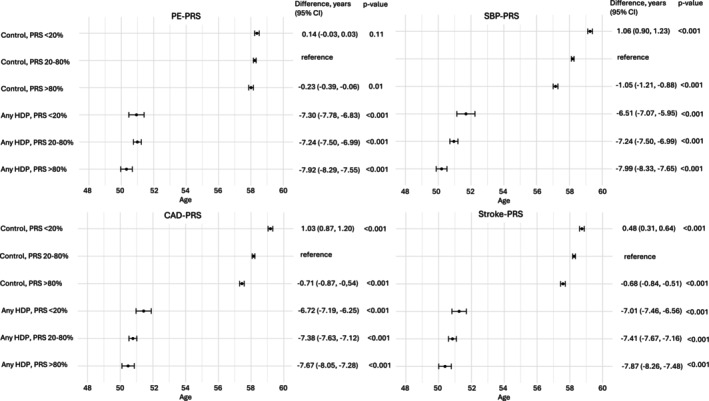
Mean age at cardiovascular disease (CVD) onset across hypertensive disorder of pregnancy (any HDP) and control groups stratified by polygenic risk score (PRS) percentiles. Women with preeclampsia (PE) were included in the any HDP group. Error bars indicate 95% confidence intervals. *p*‐values reflect two‐sided comparisons against the control group with mid‐range PRS (20%–80%). Any HDP and control women were divided according to PRSs for PE (PE‐PRS), systolic blood pressure (SBP‐PRS), coronary artery disease (CAD‐PRS) and stroke (stroke‐PRS) in the groups of low (<20th percentile), moderate (20th–80th percentile) and high risk (>80th percentile).

We also analyzed the age of CVD onset in the groups of PE and any HDP with respective control groups. We observed that women who had a history of PE or any HDP showed an earlier age of CVD onset (Figures 4 and 5). Further, we divided these women into the groups of low, moderate, and high PRS. High SBP‐PRS presented the strongest association with earlier onset of CVD in all groups. With other PRSs, the trend was not as clear. However, the history of PE and any HDP showed a stronger effect on the early onset age than any of the tested PRSs.

## 4 DISCUSSION

Our study shows that a history of PE and any HDP is associated with an increased risk of CVD in women with low, moderate, and high PE‐PRS, SBP‐PRS, CAD‐PRS, and stroke‐PRS compared to women with a history of normotensive pregnancies and respective PRS scores. The risk appears to remain elevated at least until 80 years of age. High PRS independently was associated with a minor elevation of CVD risk in women with normotensive pregnancies, but compared with the role of obstetric history, the effect appears to be mild.

Our results might suggest permanent alterations in the circulatory system predisposing women affected by PE and any HDP to CVDs. The risk of CVD in women who have the highest genetic susceptibility remains close to those with a moderate genetic risk if their pregnancies were normotensive. However, if these women are affected by PE or any HDP, the risk increases two‐fold on average. Shared genetic background is probably involved but, according to our results, does not explain the association thoroughly.

In a recent study, high SBP‐PRS was shown to be associated with a higher risk for chronic hypertension and CVD^23^. Our study shows a similar, respective risk elevation with elevating PRS score in the PE and any HDP groups, although the pregnancy history appeared to be an even more remarkable risk factor. Furthermore, our results are in agreement with those reporting an earlier age of onset in those women with high PRSs.^23^ The CVD onset age was associated with PRS levels; those with the highest PRSs were affected by CVDs at a younger age. In our data, however, pregnancy history predicted the early onset age more strongly.

The present study supports the results of Lee et al. showing a high HDP‐PRS as an independent risk factor for atherosclerotic cardiovascular diseases^24^ and the association remained even after adjusting for HDP history. Nevertheless, our results imply the role of pregnancy history to be stronger than the genetic risk itself, which in our study had only a minor independent effect.

Previous epidemiological studies have shown the highest CVD risk in women with a history of early‐onset and severe form of PE.^9^, ^25^, ^26^ Our study demonstrates a high risk of CVD in this subgroup even compared to the PE group in its entirety. However, the number of women in this subgroup was relatively small and thus the subgroup analyses should be repeated in a larger study population.

Our results further reinforce the role of PE and any HDP as major risk factors of future CVD. History of HDP is a significant risk factor, even compared to the genetic risk for CVD in women belonging to the top 20th percentile of PRS. Nevertheless, genetic susceptibility to PE, high SBP, CAD, and stroke can be identified as independent risk factors of CVD. High genetic risk for PE without hypertensive pregnancies is associated with only a minor elevation of CVD risk, and the effect was mildest of the tested PRSs. SBP‐PRS shows the strongest independent effect on the CVD risk in women with normotensive pregnancies, elevating the risk of CVD 1.3‐fold. As shown in our previous study,^27^ high SBP‐PRS is associated with elevated blood pressure levels already in early pregnancy, which might explain higher HR in this subgroup. When we excluded chronic hypertension from CVD outcomes, the association was attenuated. In a similar manner, a slight elevation of CVD risk was seen in women with high CAD‐ or stroke‐PRS.

Our results show that pregnancy can reveal important aspects of a woman's cardiovascular health that persist throughout her entire life course. Stratifying the risk for CVD after PE or any HDP should begin already after delivery to provide better targeted CVD risk management guidelines and individually tailored clinical follow‐up for primary prevention of CVD. Our results suggest the significant role of pregnancy history in the overall risk assessment even compared to comprehensive genetic information, although PRSs and other genetic examinations might in the future support the individual risk assessment of CVD and other diseases.^13^, ^15^ Even with a high genetic risk of CVD, if a woman had normotensive pregnancies, the risk of CVD seems to remain moderate until elderly. However, by the age of 80 the women most susceptible for CVD might have deceased. Possibly, if the woman lives until old age, the impact of pregnancy history might not be as crucial as other lifestyle‐related factors.

Our study has several strengths. First, our cohort is comprehensive, including the genetic information of over 213 000 women, almost 18 000 women with any HDP, of which almost 9000 women had PE. Our data reflect the general Finnish population. Additionally, Finnish registers are generally comprehensive, offering an accurate and dependable ground for research.^28^ In our study, the follow‐up time was until 60 and 80 years of age, offering a sufficient timeframe to examine the incidence of CVD. To leverage the extensive temporal coverage of the Finnish health registers, we employed ICD‐10, ICD‐9, and ICD‐8 code classifications, enabling the identification of the given diagnoses over a prolonged time span.

Limitations of this study should also be acknowledged. First, the population in Finland is genetically homogeneous compared to many other countries, and therefore generalizability to other, more diverse populations might be limited. However, as PRSs used in our study were calculated from GWAS studies in diverse populations of European, Asian, and other ancestries,^17^, ^18^, ^19^ we were able to partially mitigate concerns related to the limited genetic diversity of the Finnish population. Furthermore, in our study population, there is a possibility of underestimation of CVD diagnosis before the first pregnancy. Especially hypertensive diseases might be asymptomatic, and thus a hypertensive woman might not have a diagnosis of chronic hypertension. As is typical for large population cohorts, the limited availability of detailed clinical information restricted our ability to adjust for many potential confounders. The replication of these analyses with a smaller data set, including more specific health‐record information, is needed. Finally, the number of women in the group of PE with severe symptoms was relatively small, and the results regarding this subgroup should be interpreted with reservation. Further studies are needed to verify the results.

## 5 CONCLUSION

In this study, we observed that in women with PE or any HDP, a high genetic risk for PE, high SBP, CAD, and stroke further increased the overall risk of CVD. With normotensive pregnancies, a high genetic risk measured with PRSs caused a minor elevation in the CVD risk. Our results suggest that obstetric history is a relevant tool in evaluating a woman's long‐term risk of CVD; however, PRSs might play a role on the side of clinical risk factors. If a woman remains normotensive in her pregnancies, the high genetic risk does not seem to cause a significant increase in CVD risk. After PE or any HDP, the elevated risk seems to last at least until 80 years of age. The results highlight the importance of screening in women affected by PE or any HDP. Informing these women of the increased CVD risk would offer an opportunity to diminish the clinical risk factors that can be affected and thus reduce the overall risk of CVD. The clinical risk assessment is cost‐effective and seems superior even compared to genetic risk evaluation with PRSs.

## AUTHOR CONTRIBUTIONS

Conceptualization: Anna Kivioja, Jaakko Tyrmi, Elli Toivonen, Heini Huhtala, Tiina Jääskeläinen, Tanja Saarela, Hannele Laivuori. Data curation: Jaakko Tyrmi, FINNGEN. Formal analysis: Anna Kivioja, Jaakko Tyrmi, Heini Huhtala. Funding acquisition: Anna Kivioja, Jaakko Tyrmi, Tanja Saarela, Hannele Laivuori. Investigation: Anna Kivioja, Jaakko Tyrmi, FINNGEN, Elli Toivonen, Heini Huhtala, Tiina Jääskeläinen, Tanja Saarela, Hannele Laivuori. Methodology: Anna Kivioja, Jaakko Tyrmi, Elli Toivonen, Heini Huhtala, Tiina Jääskeläinen, Tanja Saarela, Hannele Laivuori. Project administration: Tanja Saarela, Hannele Laivuori. Resources: Jaakko Tyrmi, Tanja Saarela, Hannele Laivuori. Software: Jaakko Tyrmi. Supervision: Heini Huhtala, Tiina Jääskeläinen, Johannes Kettunen, Tanja Saarela, Hannele Laivuori. Validation: Heini Huhtala, Tiina Jääskeläinen, Johannes Kettunen, Tanja Saarela, Hannele Laivuori. Visualization: Anna Kivioja, Jaakko Tyrmi. Writing—original draft: Anna Kivioja, Jaakko Tyrmi. Writing—review and editing: Anna Kivioja, Jaakko Tyrmi, Elli Toivonen, Heini Huhtala, Tiina Jääskeläinen, Johannes Kettunen, Tanja Saarela, Hannele Laivuori.

## FUNDING INFORMATION

This project was supported by Competitive State Research Financing of the Expert Responsibility Area of Tampere University Hospital, Finska Läkaresällskapet, The Finnish Foundation for Cardiovascular Research. AK received a grant for dissertation research from the Finnish Cultural Foundation in 2022. JT is funded by EraPerMed JTC 2020, Academy of Finland (344695).

## CONFLICT OF INTEREST STATEMENT

TJ and HL: honoraria from Orion Corporation and all other authors declare no conflicts of interest.

## ETHICS STATEMENT

The Coordinating Ethics Committee of the Hospital District of Helsinki and Uusimaa (HUS) approved the FinnGen study protocol (Nr HUS/990/2017) on May 23, 2017. The FinnGen study is approved by the Finnish Institute for Health and Welfare (permit numbers: THL/2031/6.02.00/2017, THL/1101/5.05.00/2017, THL/341/6.02.00/2018, THL/2222/6.02.00/2018, THL/283/6.02.00/2019, THL/1721/5.05.00/2019, THL/1524/5.05.00/2020, and THL/2364/14.02/2020), the Digital and Population Data Service Agency (permit numbers: VRK43431/2017–3, VRK/6909/2018–3, VRK/4415/2019‐3), the Social Insurance Institution (permit numbers: KELA 58/522/2017, KELA 131/522/2018, KELA 70/522/2019, KELA 98/522/2019, KELA 138/522/2019, KELA 2/522/2020, KELA 16/522/2020) and Statistics Finland (permit numbers: TK‐53‐1041‐17 and TK‐53‐90‐20). The Biobank Access Decisions for the FinnGen samples and the data utilized in the FinnGen Data Freeze 6 include: THL Biobank BB2017_55, BB2017_111, BB2018_19, BB_2018_34, BB_2018_67, BB2018_71, BB2019_7, BB2019_8, BB2019_26, BB2020_1, Finnish Red Cross Blood Service Biobank 7.12.2017, Helsinki Biobank HUS/359/2017, Auria Biobank AB17‐5154, Biobank Borealis of Northern Finland_2017_1013, Biobank of Eastern Finland 1186/2018, Finnish Clinical Biobank Tampere MH0004, Central Finland Biobank 1–2017, and Terveystalo Biobank STB 2018001.

## Supporting information


Data S1.

**Figure S1.** Inclusion and exclusion criteria in the groups of any hypertensive disorder of pregnancy (HDP), preeclampsia (PE) and PE with severe symptoms as well as control groups according to International Classification of Diseases (ICD) ‐codes. Cardiovascular disease (CVD) outcome with ICD‐codes also defined in this figure.
**Table S1.** Hazard ratios (HR) with 95% confidence intervals (CI) calculated for the risk of cardiovascular disease (CVD) until 80 years of age and without chronic hypertension (ICD‐10, I10) as a CVD endpoint in the study groups. The groups were stratified by polygenic risk scores (PRSs) for preeclampsia, systolic blood pressure, coronary artery disease and stroke, to percentiles (low <20th percentile, moderate 20th–80th percentile, high >80th percentile). Control women with moderate PRS served as a reference group.
**Table S2.** Hazard ratios (HR) with 95% confidence intervals (CIs) calculated for the risk of cardiovascular disease (CVD) until 80 years of age and without chronic hypertension (ICD‐10, I10) as a CVD endpoint in normotensive women. Women were stratified by polygenic risk scores (PRSs) for preeclampsia, systolic blood pressure, coronary artery disease and stroke, to percentiles (low <20th percentile, moderate 20th–80th percentile, high >80th percentile). Women with moderate PRS served as a reference group.

## Data Availability

The data that support the findings of this study are available from the corresponding author upon reasonable request.
