# Supplementary material for: Long‐term cardiovascular risk in women with hypertensive disorders of pregnancy: Insights from polygenic risk scores
Source: Acta Obstet Gynecol Scand. 2025 Jul 31;104(10):1907–17. doi: 10.1111/aogs.70021 (PMC12451206; doi:10.1111/aogs.70021)
Supplement: Supplementary file 2 — Figure S1. Inclusion and exclusion criteria in the groups of any hypertensive disorder of pregnancy (HDP), preeclampsia (PE) and PE with severe symptoms as well as control groups according to International Classification of Diseases (ICD) ‐codes. Cardiovascular disease (CVD) outcome with ICD‐codes also defined in this figure. Table S1. Hazard ratios (HR) with 95% confidence intervals (CI) calculated for the risk of cardiovascular disease (CVD) until 80 years of age and without chronic hypertension (ICD‐10, I10) as a CVD endpoint in the study groups. The groups were stratified by polygenic risk scores (PRSs) for preeclampsia, systolic blood pressure, coronary artery disease and stroke, to percentiles (low <20th percentile, moderate 20th–80th percentile, high >80th percentile). Control women with moderate PRS served as a reference group. Table S2. Hazard ratios (HR) with 95% confidence intervals (CIs) calculated for the risk of cardiovascular disease (CVD) until 80 years of age and without chronic hypertension (ICD‐10, I10) as a CVD endpoint in normotensive women. Women were stratified by polygenic risk scores (PRSs) for preeclampsia, systolic blood pressure, coronary artery disease and stroke, to percentiles (low <20th percentile, moderate 20th–80th percentile, high >80th percentile). Women with moderate PRS served as a reference group. [file AOGS-104-1907-s001.docx]

Table S1. Hazard ratios (HR) with 95% confidence intervals (CI) calculated for the risk of cardiovascular disease (CVD) until 80 years of age and without chronic hypertension (ICD-10, I10) as a CVD endpoint in the study groups. The groups were stratified by polygenic risk scores (PRSs) for preeclampsia, systolic blood pressure, coronary artery disease and stroke, to percentiles (low <20^th^ percentile, moderate 20-80^th^ percentile, high >80^th^ percentile). Control women with moderate PRS served as a reference group.

| Study phenotype | Any hypertensive disorder of pregnancy  n= 17 916 | | | |  | Preeclampsia  n= 8858 | | | |  | Preeclampsia with severe symptoms  n=1811 | | | |
| --- | --- | --- | --- | --- | --- | --- | --- | --- | --- | --- | --- | --- | --- | --- |
| PRS  % group | HR, no I10 | p-value | HR, until 80 y | p-value |  | HR, no I10 | p-value | HR, until 80 y | p-value |  | HR, no I10 | p-value | HR, until 80 y | p-value |
| **Preeclampsia** |  |  |  |  |  |  |  |  |  |  |  |  |  |  |
| <20 | 1.45 | 2x10^-11^ | 1.61 | 2x10^-16^ |  | 1.34 | 2x10^-4^ | 1.46 | 1x10^-12^ |  | 1.82 | 6x10^-5^ | 1.98 | 2x10^-10^ |
| 20-80 | 1.55 | 2x10^-16^ | 1.71 | 2x10^-16^ |  | 1.51 | 2x10^-16^ | 1.62 | 2x10^-16^ |  | 1.72 | 4x10^-12^ | 1.85 | 2x10^-16^ |
| >80 | 1.65 | 2x10^-16^ | 1.89 | 2x10^-16^ |  | 1.52 | 2x10^-13^ | 1.79 | 2x10^-16^ |  | 1.52 | 0.001 | 1.87 | 4x10^-13^ |
| **Systolic blood pressure** |  |  |  |  |  |  |  |  |  |  |  |  |  |  |
| <20 | 1.47 | 1x10^-9^ | 1.28 | 6x10^-8^ |  | 1.38 | 1x10^-4^ | 1.23 | 6x10^-4^ |  | 1.52 | 0.02 | 1.59 | 3x10^-4^ |
| 20-80 | 1.55 | 2x10^-16^ | 1.67 | 2x10^-16^ |  | 1.49 | 2x10^-16^ | 1.58 | 2x10^-16^ |  | 1.81 | 4x10^-14^ | 1.90 | 2x10^-16^ |
| <80 | 1.66 | 2x10^-16^ | 2.14 | 2x10^-16^ |  | 1.57 | 2x10^-16^ | 2.04 | 2x10^-16^ |  | 1.59 | 7x10^-5^ | 2.02 | 2x10^-16^ |
| **Coronary artery disease** |  |  |  |  |  |  |  |  |  |  |  |  |  |  |
| <20 | 1.35 | 1x10^-7^ | 1.55 | 2x10^-16^ |  | 1.30 | 6x10^-8^ | 1.45 | 2x10^-13^ |  | 1.66 | 0.002 | 1.58 | 2x10^-4^ |
| 20-80 | 1.57 | 2x10^-16^ | 1.75 | 2x10^-16^ |  | 1.50 | 2x10^-16^ | 1.65 | 2x10^-16^ |  | 1.71 | 4x10^-12^ | 1.95 | 2x10^-16^ |
| >80 | 1.77 | 2x10^-16^ | 1.93 | 2x10^-16^ |  | 1.64 | 2x10^-16^ | 1.79 | 2x10^-16^ |  | 1.71 | 9x10^-6^ | 1.92 | 7x10^-14^ |
| **Stroke** |  |  |  |  |  |  |  |  |  |  |  |  |  |  |
| <20 | 1.50 | 6x10^-15^ | 1.56 | 2x10^-16^ |  | 1.47 | 4x10^-8^ | 1.46 | 2x10^-14^ |  | 1.65 | 6x10^-4^ | 1.73 | 3x10^-7^ |
| 20-80 | 1.55 | 2x10^-16^ | 1.76 | 2x10^-16^ |  | 1.44 | 2x10^-16^ | 1.60 | 2x10^-16^ |  | 1.65 | 5x10^-10^ | 1.80 | 2x10^-16^ |
| >80 | 1.73 | 2x10^-16^ | 1.87 | 2x10^-16^ |  | 1.69 | 2x10^-16^ | 1.89 | 2x10^-16^ |  | 1.94 | 9x10^-8^ | 2.24 | 2x10^-16^ |

PRS; polygenic risk score, HR; hazard ratio.

Table S2. Hazard ratios (HR) with 95% confidence intervals (CI) calculated for the risk of cardiovascular disease (CVD) until 80 years of age and without chronic hypertension (ICD-10, I10) as a CVD endpoint in normotensive women. Women were stratified by polygenic risk scores (PRSs) for preeclampsia, systolic blood pressure, coronary artery disease and stroke, to percentiles (low <20^th^ percentile, moderate 20-80^th^ percentile, high >80^th^ percentile). Women with moderate PRS served as a reference group.

| Normotensive n= 196 026 | | | | | | | |
| --- | --- | --- | --- | --- | --- | --- | --- |
| PRS | HR, no I10 |  | p-value |  | HR, until 80 y |  | p-value |
| % group |  |  |  |  |  |  |  |
| **Preeclampsia** |  |  |  |  |  |  |  |
| <20 | 0.99 |  | 0.46 |  | 0.98 |  | 0.15 |
| 20-80 | 1 |  |  |  | 1 |  |  |
| >80 | 1.04 |  | 0.10 |  | 1.02 |  | 0.05 |
| **Systolic blood pressure** |  |  |  |  |  |  |  |
| <20 | 0.90 |  | 1x10^-7^ |  | 0.90 |  | 8x10^-15^ |
| 20-80 | 1 |  |  |  | 1 |  |  |
| >80 | 1.16 |  | 2x10^-16^ |  | 1.17 |  | 2x10^-16^ |
| **Coronary artery disease** |  |  |  |  |  |  |  |
| <20 | 0.91 |  | 4x10^-7^ |  | 0.90 |  | 2x10^-16^ |
| 20-80 | 1 |  |  |  | 1 |  |  |
| >80 | 1.17 |  | 2x10^-16^ |  | 1.17 |  | 2x10^-16^ |
| **Stroke** |  |  |  |  |  |  |  |
| <20 | 0.94 |  | 0.002 |  | 0.92 |  | 2x10^-13^ |
| 20-80 | 1 |  |  |  | 1 |  |  |
| >80 | 1.14 |  | 4x10^-14^ |  | 1.14 |  | 2x10^-16^ |

PRS; polygenic risk score.

Figure S1. Inclusion and exclusion criteria in the groups of hypertensive disorder of pregnancy (any HDP), preeclampsia (PE) and PE with severe symptoms and as well as control groups according to International Classification of Diseases (ICD) -codes. Cardiovascular disease (CVD) outcome with ICD-codes also defined in this figure.
